# The Antimicrobial Effect of Lactobacillus Casei Culture Supernatant Against Multiple Drug Resistant Clinical Isolates of Shigella Sonnei and Shigella Flexneri in Vitro

**DOI:** 10.5812/ircmj.7454

**Published:** 2013-02-05

**Authors:** Reza Mirnejad, Ali Reza Vahdati, Jamal Rashidiani, Mohammad Erfani, Vahhab Piranfar

**Affiliations:** 1 Molecular Biology Research Center, Baqiyatallah University of Medical Sciences, Tehran, IR Iran; 2 Institute of Evolutionary Biology and Environmental Studies, University of Zurich, Zurich, Switzerland; 3 Nano Biotechnology Research Center, Baqiyatallah University of Medical Sciences, Tehran, IR Iran; 4 Department of Microbiology, Tonekabon Branch, Islamic Azad University of Tonekabon, Tonekabon, IR Iran

**Keywords:** Lactobacillus Casei, Shigella Flexneri, Shigella Sonnei

## Abstract

**Backgrounds:**

Shigellosis remains an important public health problem in developing countries with S. sonnei and S. flexneri in US, Europe and in Asian countries being of importance.

**Objectives:**

This study evaluates the protective effect of Lactobacillus casei cell-free culture supernatants (CFCS) against multiple drug resistance (MDR) clinical samples of Shigella sonnei and Shigella flexneri in vitro.

**Materials and Methods:**

S. sonnei and S .flexneri was identified by common microbiological and serological methods. Antibiogram with 18 antibiotics were tested for 34 positive cultures by disc diffusion method. The Samples showed considerable resistance to antibiotics. Antimicrobial effects of CFCS were tested against S. sonnei and S. flexneri by agar-well assay and broth micro dilution methods. In addition, the antimicrobial activity remained active treatment after adjust pH 7, adding Proteinase K and heating for L. casei.

**Results:**

The results implicate that L. casei strongly inhibits the development of pathogen samples. In contrast, via the disc diffusion method 4 out of 18 antibiogram have shown complete resistance against the pathogen samples. In addition, the natures of antimicrobial properties have been tested in different conditions such as various pH, temperature and presence of proteinase K. The MIC50 (minimum inhibitory concentration) and MIC90 of CFCS of L. casei were determined, for S. sonnei were 2.25 and 10.5, for S .flexneri were 5.25 and 5.25 respectively. The results have shown a significant resistance pattern by these four antibiotics in this case.

**Conclusions:**

The data indicates that. L. casei highly resistant against to antibiotics, heat, Proteinase K and so many activities against MDR Shigella pathogenic strains . L. casei is the best probiotics candidate.

## 1. Background

Shigellosis remains an important public health problem in developing countries with S. sonnei and S. flexneri in US, Europe and in Asian countries being of importance (1-4). Shigella is one of the most antimicrobial-resistant bacteria (3, 5) and an important causeof gastroenteritis-induced deaths in 3-5 million children aged less than five years in developing countries (6, 7). Shigella ranks the third among bacterial food borne pathogens (after Campylobacter and Salmonella) in the number of gastrointestinal cases according to the report of Centers for Disease Control and Prevention (8, 9). The emergence of multiple drug resistance to cost-effective antimicrobials against Shigella is a matter of concern in developing countries and resistant pattern of these bacteria is the cause of numerous clinical problems throughout the world. Increased resistance among pathogens causing nosocomial and community acquired infections is known to related to the widespread utilization of antibiotics (10). Preparing the prevention and treatment protocols with natural patterns in this regard seems to be necessary (11). Recent reports have documented the role of Lactobacillus in prevention and treatment of some infections. Lactobacillus strains have commensally in the human body (12). Its beneficial effect may be associated to its ability to inhibit the growth of pathogens, apparently by the secretion of antibacterial substances including lactic acid, hydrogen peroxide and etc. (13) Now, the application of probiotics to the prevention and management of gastrointestinal disorders has received much interest (5). The antimicrobial activity of a wide range of pathogenic microorganisms by Lactobacillus either in vitro or in vivo has been reported, including E. coli, Salmonella, Pseudomonas aeruginosa and Staphylococcus aureus (1, 14-16). However, the antimicrobial activity against S. flexneri and S. sonnei has been little known. So the main objective of this study was to apply establishes in vitro tests to evaluate the nature of antimicrobial substances and antimicrobial properties of L. casei against multi-drug resistant clinical isolates of S. flexneri and S. sonnei. Furthermore, we evaluated the tolerance properties to confirm the tested L. casei supernatant could be potentially used as probiotic.

## 2. Objectives

This study evaluates the protective effect of Lactobacillus casei cell-free culture supernatants (CFCS) against multiple drug resistance (MDR) clinical samples of Shigella sonnei and Shigella flexneri in vitro.

## 3. Materials and Methods

### 3.1. Bacterial Strains and Culture Conditions

Clinical specimens (S. flexneri and S. sonnei) were collected from suspicious patients with clinical history of Shigellosis. Stool specimens were plated into plate Shigella Salmonella agar (Hi-Media, Mumbai, India) and deoxycholate citrate agar (DCA) (Hi-Media, Mumbai, India) as primary plates and a loop full also inoculated to Selenite Broth (Selenite F Broth) (Hi-Media, Mumbai, India). Suspicious colonies on plates, which were oxidase test negative and Gram negative, were biochemically characterized (5). Lactobacillus casei was provided by Microbiological Laboratory of Clinic Detection Center of IRAN (Tehran, IRAN).

### 3.2. Susceptibility Testing

Susceptibility of S. flexneri and S. sonnei to 18 antibiotics including, ampicillin, ceftriaxon, chloramphenicol, cefotaxime, ceftazidime, tobramycin, kanamycin, co-amoxiclav, ticarcillin, nalidixic acid, ciprofloxacin, tetracycline, chlorotetracycline, streptomycin, cephalothin, trimethoprim/sulfamethoxazole, gentamicin and amikacin were investigated by using Kirby-Bauer disk diffusion method and comparing their growth inhibition zones to those reported by CLSI (5, 17). The diameters of inhibition zones were measured and compared with the zones suggested by CLSI, using susceptible strains as control. From these isolates, 34 sample were selected for mix by supernatant L. casei culture test.

### 3.3. Antimicrobial Activity and Nature of Antimicrobial Substances

The inhibitory activity of supernatants of L. casei was screened against multiple drug resistant Shigella isolates using conventional Agar-well assay. Cell-free culture supernatants (CFCS) were obtained by centrifugation (13,000 ×g, 4 ◦C, 15 min) of L. casei cultures grown in 20 ml MRS broth at 37 ◦C for 24 h. The supernatant was filtered through a 0.22 mm filter to remove cells, and then 1 ml CFCS of L. casei was retained as untreated filtrate. To determine the organic acid function, 1 ml CFCS was adjusted to pH 7. In order to test the heat sensitivity, 1 ml CFCS of the L. casei were incubated at 100 ◦C for 15 min. Proteinase K sensitivity was evaluated by incubating 1 ml CFCS with a final concentration of 1 mg/ml Proteinase K at 37 ◦C for 4 h. The antimicrobial activity of samples was tested using the Agar-well assay (18). Briefly, indicator bacteria were grown in TSB broth overnight and spread onto the TSA agar plate after diluting to 107 CFU/ml, then 5 mm-diameter wells were punched into the surface using a sterile borer. Subsequently, 50 µl samples prepared from filtrate was added to each well of the plate and incubated at 37 ◦C for 24 hrs. The antimicrobial activity was recorded as growth free inhibition zones (diameter) around the well. MRS adjusted at pH 7 served as control.

### 3.4. Determination of Minimum Inhibitory Concentration (Mic)

Minimum concentration of L. casei inhibitory to the growth of 50 per cent (MIC50) and 90 per cent (MIC90) of the isolates was determined on mueller-hinton agar (MHA) in a 9 cm plate. The agar contained concentration ranges of L. casei prepared by two-fold serial dilution according to the National Committee for Clinical Laboratory Standards (NCCLS). Manual inoculation with micropipette for dispensing 20 µl of standardized inoculum (10 ml) of each isolate onto the surface of L. casei plate was done to obtain a final inoculum size of 104-105cfu/spot. CFCS free plates were inoculated at the end and were used as negative controls. The positive controls were the plates inoculated with the reference strain. MIC50 and MIC90 of each antimicrobial agent against Shigella isolates were evaluated after incubating the plates, containing completely absorbed inoculum, in ambient air at 37◦C for 24 h (19).

### 3.5. Turbidimetry

One ml of supernatants of L. casei was mixed with 1 ml of the MDR Shigella strains cultures (4 × 105 CFU/ml) in mueller-hinton broth. The optical densities of culture media were measured at 0, 6, 12, 18 and 24 hrs after incubation at 580 nm. Also the CFU were counted by the spread-plate technique.

### 3.6. Statistical Analysis

All experiments were performed three times independently and each assay was performed in duplicate. Results were expressed as means ± standard deviation. The level of significance was analyzed by chi-square test and ANOVA (P < 0.05) using SPSS 17.0 for Windows (SPSS Inc.).

## 4. Results 

**Figure 1:**
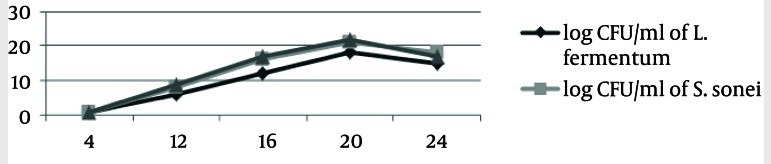
Growth Curve of Bacteria in Four Separate

**Figure 2:**
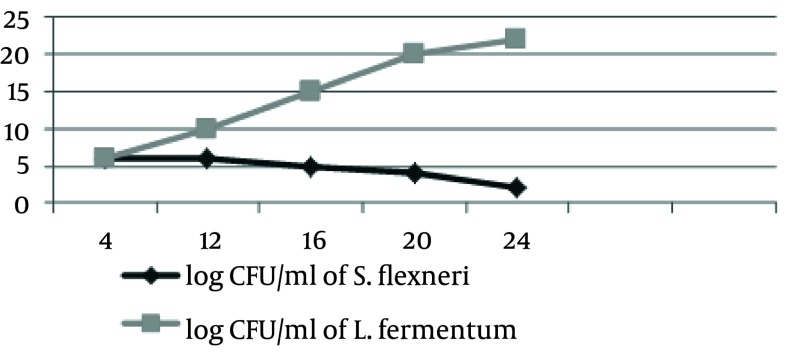
S.flexneri Growth Curve Presence L. Fermentum

**Figure 3:**
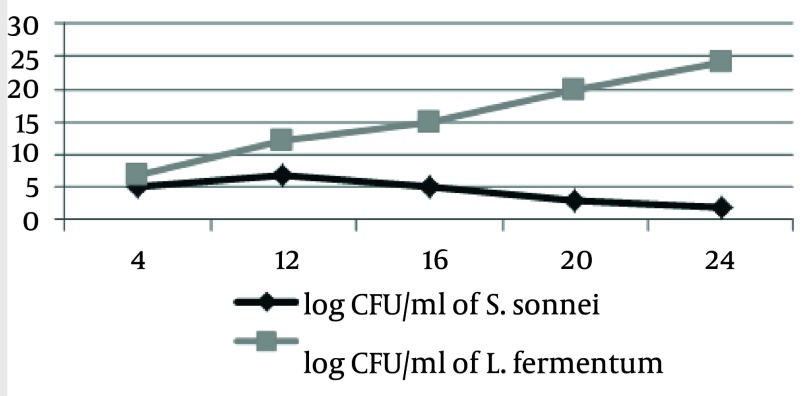
S.sonnei Growth Curve Presence L. fermentum

S. sonnei and S .flexneri was isolated from clinical samples, and confirmed by phenotypic and genotypic methods. Thirty four Shigella were isolated from stool samples. Of these, S. flexneri 21 (61.77%) was the most common isolate in all age groups, followed by S. sonnei 13 (38.23%). CFCS of supernatant of L. casei displayed antimicrobial activity against 34 samples of S. sonnei and S. flexneri by the agar-well assay, respectively. These L. casei showed high levels of antimicrobial activity against S. sonnei and S. flexneri. When the CFCS was adjusted to pH 7, the antimicrobial activity was abolished. In addition, the antimicrobial activity remained active after Proteinase K and heat treatment for L. casei as shown in Table 1. The resistant pattern of 34 Shigella to antimicrobial agents is shown in Table 2. Moreover, all tested strains showed resistance against tetracycline, streptomycin, trimethoprim/sulfamethoxazole and ampicillin. The spread of antibiotic resistance for other potent antimicrobial agents is also shown (Table 2). The average MIC50 and MIC90 for Lactobacillus casei on S. flexneri and S. sonnei, respectively, 5.25, 5.25, 2.25 and 10.5 were recorded. The MIC tests were repeated for each Shigella isolates at least three times. The turbidity method survey, optimal growth was observed for standard curve of growth in the control group with L. casei and Shigella isolates in standard conditions (Figure 1). The second group exposed to L. casei supernatant with Shigella isolates. The Growth of pathogenic bacteria appeared to be less significant (Figures 2 and 3).

**Table 1:** The Antimicrobials Activity of L. casei CFCS with Several Treatments

Indicator strains	Inhibition zone, mm^a^			
	Non-treatment of CFCS	Proteinase K	CFCS adjust pH 7	Heat (100 ◦ C, 15 min)
	L.casei	L.casei	L.casei	L.casei
**S.sonnei**	+++	+++	-	++
**S.flexneri**	++	++	-	++

^a^Symbols refer to the size of the inhibition zone diameter observed with growing cells: +, 1 mm; ++, 2 mm; +++, 2-5 mm; -, absence of an inhibitory zone.

**Table 2:** Resistance Percentage of Shigella Isolates to Various Antimicrobial Agents (Total population = 34)

Antibiotics	ShigellaIsolates		
	Resistant, No. (%)	Intermediate, No. (%)	Susceptible, No. (%)
**Ampicillin (20μg)**	34 (100)	0 (0)	0 (0)
**Tetracycline (30μg)**	34 (100)	0 (0)	0 (0)
**Streptomycin (30μg)**	34 (100)	0 (0)	0 (0)
**Trimethoprim/sulfamethoxazole(1.25/23.75μg)**	34 (100)	0 (0)	0 (0)
**Ciprofloxacin (5μg)**	0 (0)	34 (100)	0 (0)
**Chlorotetracycline(30μg)**	0 (0)	34 (100)	0 (0)
**Nalidixicacid (30μg)**	13 (38.24)	0 (0)	21 (61.76)
**Co-amoxiclav(20/10μg)**	10 (29.41)	14 (41.17)	10 (29.41)
**Chloramphenicol (30μg)**	7 (29.59)	0 (0)	27 (79.41)
**Tobramycin (10μg)**	6 ( 17.65)	0 (0)	28 (82.35)
**Amikacin(30μg)**	5 (14.71)	5 (14.71)	24 (70.58)
**Cefotaxime(30μg)**	3 (8.83)	30 (88.23)	1 (2.94)
**Cephalothin(30μg)**	3 (8.83)	1 (2.94)	30 (88.23)
**Ceftriaxon(30μg)**	3 (8.83)	0 (0)	31 (91.7)
**Ticarcillin(75μg)**	3 (8.83)	0 (0)	31 (91.7)
**Kanamycin (30μg)**	1 (~ 3)	16 (47)	17 (50)
**Ceftazidime(30μg)**	1 (2.94)	1 (2.94)	32 (94.12)
**Gentamicin(10μg)**	1 (2.94)	1 (2.94)	32 (94.12)

## 5. Discussion

S. sonnei and S. flexneri, are entero-invasive, cause the inflammatory destruction of the intestinal epithelium (20), leading to an acute recto-colitis causing lethal complications. High prevalence of Shigella with multiple antibiotic resistance isolates was observed in this study. None of the antimicrobial agents was effective against all the multi-drug tested strains demonstrating the current problem in the treatment of multiple drug resistant nosocomial infections. Shigella isolates strains showed complete resistance against tetracycline, streptomycin, trimethoprim/sulfamethoxazole and ampicillin. Furthermore, its susceptibility to other potent antimicrobial agents, including ciprofloxacin, chloramphenicol, cefotaxime, ceftazidime, tobramycin, co-amoxiclav kanamycin, gentamicin and amikacin, which are used was also tested. Folster et al. reported decreased susceptibility to ciprofloxacin among Shigella Isolates in the United States, 2006 to 2009 (21). This study showed that all MDR samples were not resistant to ciprofloxacin in IRAN. As a functional probiotic, anti-pathogen activity is one of the important properties. The inhibitory activity of lactic acid bacteria against some resistant clinical isolates of Shigella has been reported. The L. casei tested during this study showed strong antimicrobial activity against S. sonnei and S. flexneri. However, such activity disappeared when the pH of the CFCS was adjusted to 7, and the addition of Proteinase K did not affect on the size of the inhibition zones, which indicated that bacteriocins were not involved in the antimicrobial activity of L. casei, so this activity was attributed to the production of organic acids. Toba et al. adjacent culture supernatant of different Lactobacillus with pathogenic bacteria such as Listeria monocytogenes, Salmonella and staphylococcus aureus and examined obtained Turbidity at different time intervals and concluded that turbidity levels have been studied are reduced dramatically (22). The present study also metabolites of Lactobacillus casei in presence of Pathogenic strains obtained from Clinical samples were exposed that descending of Turbidity is confirmed the above study suggests and shows that metabolite have been prevented the growth of pathogenic bacteria. In other study, Hirano et al. reviewed the antimicrobial effect of culture supernatant of lactobacillus plantarum, lactobacillus sik and lactobacillus kratos and have been in plate with technique WDA and found that these substances with the creation of inhibition zone on a wide range of pathogenic bacteria such as Yersinia enterocolitica and Listeria have inhibitory effect (23). In the present study L. casei with well plate technique, against clinical samples of S. sonnei and S. flexneri showed antimicrobial activity and inhibition zone related metabolites of Lactobacillus against human pathogenic strains consideration was given. In the present study, the stability of antimicrobial metabolites of L. casei against the temperature was studied and it was found thatuntil 15 minutes at 100 ° C is stable and metabolites property is maintained.

## References

[1] Gopal Pramod K, Prasad Jaya, Smart John, Gill Harsharanjit S (2001). In vitro adherence properties of Lactobacillus rhamnosus DR20 and Bifidobacterium lactis DR10 strains and their antagonistic activity against an enterotoxigenic Escherichia coli.. International Journal of Food Microbiology..

[2] Gu B, Cao Y, Pan S, Zhuang L, Yu R, Peng Z (2012). Comparison of the prevalence and changing resistance to nalidixic acid and ciprofloxacin of Shigella between Europe-America and Asia-Africa from 1998 to 2009.. Int J Antimicrob Agents..

[3] Pazhani GP, Niyogi SK, Singh AK, Sen B, Taneja N, Kundu M (2008). Molecular characterization of multidrug-resistant Shigella species isolated from epidemic and endemic cases of shigellosis in India.. J Med Microbiol..

[4] Toba Takahiro, Yoshioka Emiko, Itoh Takatoshi (1991). Acidophilucin A, a new heat-labile bacteriocin produced by Lactobacillus acidophilus LAPT 1060.. Letters in Applied Microbiology..

[5] Opintan J, Newman MJ (2007). Distribution of serogroups and serotypes of multiple drug resistant Shigella isolates.. Ghana Med J..

[6] Mandomando I, Jaintilal D, Pons MJ, Valles X, Espasa M, Mensa L (2009). Antimicrobial susceptibility and mechanisms of resistance in Shigella and Salmonella isolates from children under five years of age with diarrhea in rural Mozambique.. Antimicrob Agents Chemother..

[7] Sivapalasingam S, Nelson JM, Joyce K, Hoekstra M, Angulo FJ, Mintz ED (2006). High prevalence of antimicrobial resistance among Shigella isolates in the United States tested by the National Antimicrobial Resistance Monitoring System from 1999 to 2002.. Antimicrob Agents Chemother..

[8] Nader de Macias ME, Apella MC, Romero NC, Gonzalez SN, Oliver G (1992). Inhibition of Shigella sonnei by Lactobacillus casei and Lact. acidophilus.. J Appl Bacteriol..

[9] Zhang Y, Zhang L, Du M, Yi H, Guo C, Tuo Y (2011). Antimicrobial activity against Shigella sonnei and probiotic properties of wild lactobacilli from fermented food.. Microbiol Res..

[10] Wilson G, Easow JM, Mukhopadhyay C, Shivananda PG (2006). Isolation & antimicrobial susceptibility of Shigella from patients with acute gastroenteritis in Western Nepal.. Indian J Med Res..

[11] Mirnejad R, Jafari H, Ardebilli A, Babavalian H (2012). Reduction of Enterotoxigenic Escherichia coli colonization by the oral administration of Lactobacillus casei as a Probiotic in a Murine Model.. African J Microb Res..

[12] Jamalifar H, Rahimi H, Samadi N, Shahverdi A, Sharifian Z, Hosseini F (2011). Antimicrobial activity of different Lactobacillus species against multi- drug resistant clinical isolates of Pseudomonas aeruginosa.. Iran J Microbiol..

[13] Bhattacharya D, Purushottaman SA, Bhattacharjee H, Thamizhmani R, Sudharama SD, Manimunda SP (2011). Rapid emergence of third-generation cephalosporin resistance in Shigella sp. isolated in Andaman and Nicobar Islands, India.. Microb Drug Resist..

[14] Opintan J, Newman MJ (2007). Distribution of serogroups and serotypes of multiple drug resistant Shigella isolates.. Ghana Med J..

[15] Karska-Wysocki B, Bazo M, Smoragiewicz W (2010). Antibacterial activity of Lactobacillus acidophilus and Lactobacillus casei against methicillin-resistant Staphylococcus aureus (MRSA).. Microbiol Res..

[16] Makras L, Triantafyllou V, Fayol-Messaoudi D, Adriany T, Zoumpopoulou G, Tsakalidou E (2006). Kinetic analysis of the antibacterial activity of probiotic lactobacilli towards Salmonella enterica serovar Typhimurium reveals a role for lactic acid and other inhibitory compounds.. Res Microbiol..

[17] Saxena S, Dutta R (2011). Antimicrobial resistance pattern of Shigella species over five years at a tertiary-care teaching hospital in north India.. J Health Popul Nutr..

[18] Panhotra BR, Saxena AK, Al-Mulhim K (2004). Emergence of nalidixic acid resistance in Shigella sonnei isolated from patients having acute diarrheal disease: report from eastern province of Saudi Arabia.. Jpn J Infect Dis..

[19] Rammelsberg M, Radler F (1990). Antibacterial polypeptides of Lactobacillus species.. J Applied Microb..

[20] Zaika LL, Phillips JG (2005). Model for the combined effects of temperature, pH and sodium chloride concentration on survival of Shigella flexneri strain 5348 under aerobic conditions.. Int J Food Microbiol..

[21] Sansonetti PJ (2006). Rupture, invasion and inflammatory destruction of the intestinal barrier by Shigella: the yin and yang of innate immunity.. Can J Infect Dis Med Microbiol..

[22] Folster JP, Pecic G, Bowen A, Rickert R, Carattoli A, Whichard JM (2011). Decreased susceptibility to ciprofloxacin among Shigella isolates in the United States, 2006 to 2009.. Antimicrob Agents Chemother..

[23] Traa BS, Walker CL, Munos M, Black RE (2010). Antibiotics for the treatment of dysentery in children.. Int J Epidemiol..

[24] Hirano J, Yoshida T, Sugiyama T, Koide N, Mori I, Yokochi T (2003). The effect of Lactobacillus rhamnosus on enterohemorrhagic Escherichia coli infection of human intestinal cells in vitro.. Microbiol Immunol..

